# Supplementary material for: The BK Channel Limits the Pro-Inflammatory Activity of Macrophages
Source: Cells. 2024 Feb 9;13(4):322. doi: 10.3390/cells13040322 (PMC10886595; doi:10.3390/cells13040322)
Supplement: Supplementary file 1 [file cells-13-00322-s001.zip › cells-2837643-supplementary.pdf]

Supplementary materials

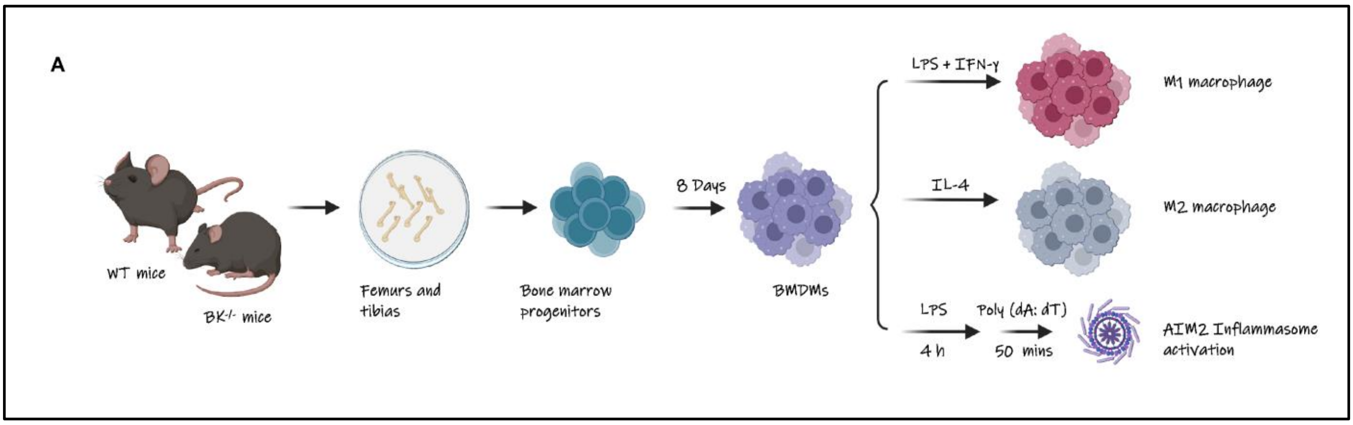

Supplementary Figure S1. Schematic presentation of BMDMs generation, polarization, and AIM2 inflammasome activation. (**A**) Untreated BMDMs, introduced as M0 macrophages, were cultured as control cells. BMDMs were polarized into M1 macrophages through stimulation with LPS (100 ng/ml) and IFN-γ (20 nM), while polarization to M2 macrophages was achieved with IL-4 (20 nM). BMDMs were primed with LPS (100 ng/ml) and subsequently stimulated with Poly(dA:dT) (1 µg/ml) to induce AIM2 inflammasome activation. Created with BioRender.com.

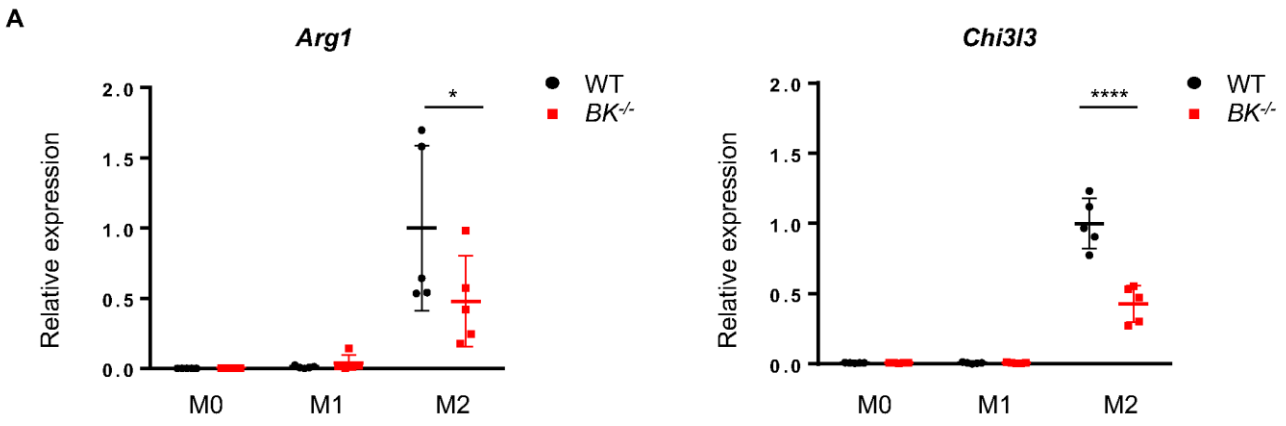

Supplementary Figure S2. The absence of the BK channel diminishes mRNA levels of the M2 macrophage markers *Arg1* and *Chi3l3*. (**A**) BMDMs were polarized to M1 and M2 macrophages for 24 h, with untreated M0 macrophages cultured as a control. Cells were lysed with RNA lysis buffer, and RNA extraction was performed. Subsequently the gene expression of the M2 macrophage markers *Arg1* and *Chi3l3* was analyzed by qPCR. Values are means ± SD. **p* < 0.05, *****p* < 0.0001. Each graph represents data from five independent experiments.

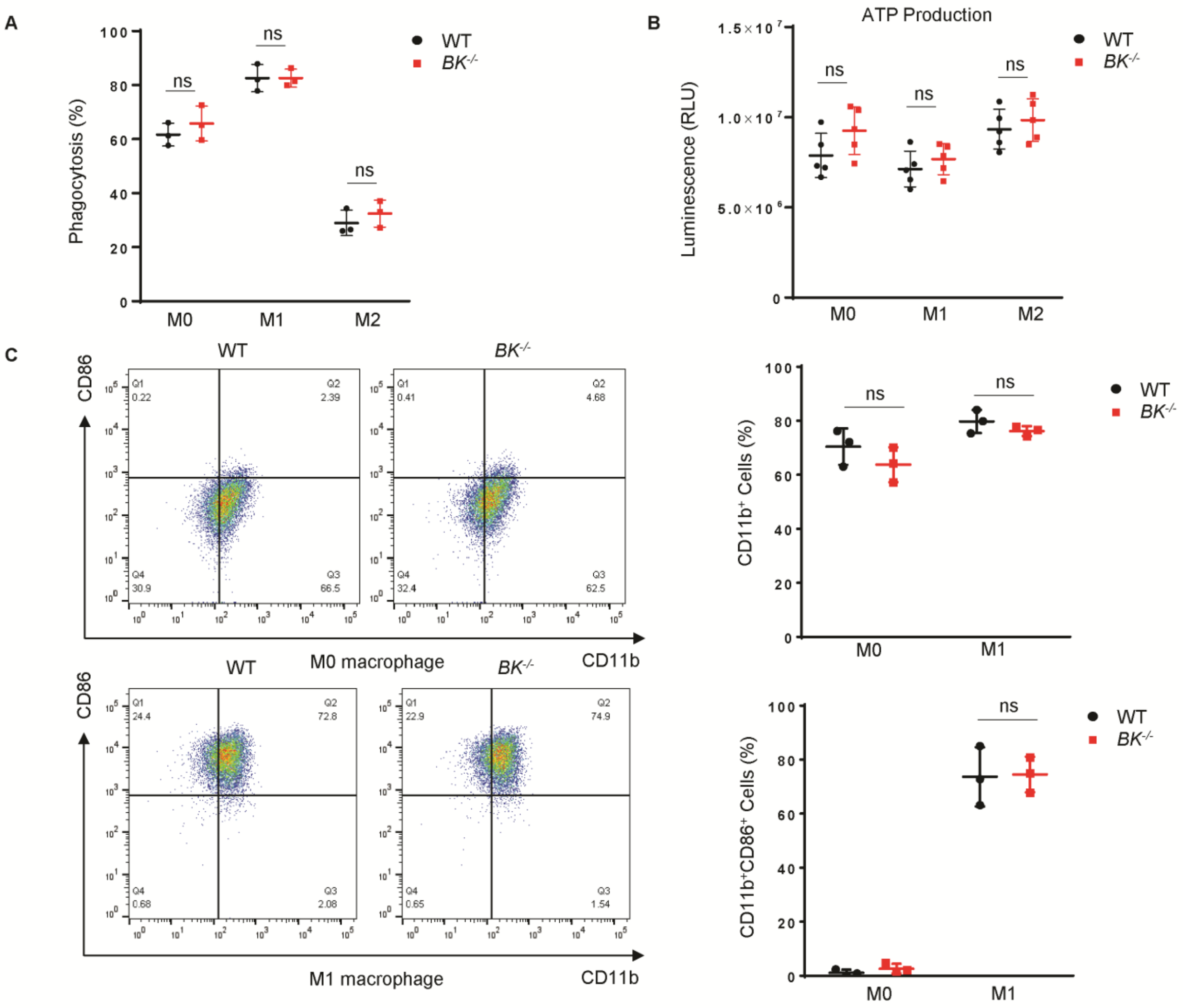

Supplementary Figure S3. The absence of the BK channel does not impact macrophage phagocytosis, ATP production, or the expression of the M1 macrophage surface marker CD86. (A) BMDMs were polarized into M1 and M2 macrophages for 24 h, with untreated M0 macrophages cultured as controls. GFP-*E. coli* was introduced to the cells at a 10-fold concentration for 30 min. Macrophage phagocytosis was assessed using flow cytometry. Values are means ± SD. ns, not significant; (B) ATP production was quantified by luminescent signal for luciferase activity in M0, M1, and M2 macrophages after 24 h of polarization. Values are means ± SD. ns, not significant; (C) BMDMs were polarized into M1 macrophages for 24 h, with untreated M0 macrophages cultured as controls. Cells were stained for the macrophage surface marker CD11b, and the macrophage population was gated. The expression of the M1 macrophage surface marker CD86 was evaluated by flow cytometry. Values are means ± SD. ns, not significant. Each graph represents data from three independent experiments.

Supplementary Table S1. List of reagents and materials.

Key Reagent and Material	SOURCE	IDENTIFIER
**Macrophage culture**		
Iscove's modified Dulbecco's medium (IMDM)	Sigma-Aldrich	Cat. #I3390
GlutaMAX™ Supplement	ThermoFisher Scientific	Cat. #35050061
Penicillin-Streptomycin	Gibco	Cat. #15140122
2-Mercaptoethanol	Gibco	Cat. #31350010
**Macrophage polarization**		
LPS	Sigma-Aldrich	Cat. #L6529
IFN-γ	Peprotech	Cat. #315-05
IL-4	Peprotech	Cat. #214-14
**Phagocytosis**		
Luria broth base (LB) medium	Sigma-Aldrich	Cat. #L7275
Not treated 12-well plate	Corning	Cat. #351143
**Inflammasome activation**		
Ultrapure LPS	Invivogen	Cat. #tlrl-3pelps
Nigericin	Sigma-Aldrich	Cat. #N7143
ATP	Invivogen	Cat. #tlrl-atpl
Poly(dA:dT)	Invivogen	Cat. #tlrl-patn
Ultrapure flagellin	Invivogen	Cat. #tlrl-epstfla
Lipofectamine 2000 Transfection Reagent	Invitrogen	Cat. #11668019
Opti-MEM	Thermo	Cat. #31985070
DOTAP Liposomal Transfection Reagent	Roche	Cat. #11202375001
**Immunoblot**		
Tris	Sigma-Aldrich	Cat. #T6066
NaCl	Sigma-Aldrich	Cat. #S9888
Glycerol	Sigma-Aldrich	Cat. #G5516
Triton X-100	Millipore	Cat. #108603
EDTA	Sigma-Aldrich	Cat. #03677
NaPyrophosphate	Sigma-Aldrich	Cat. #71501
NaF	Sigma-Aldrich	Cat. #71519
Na_3VO_4	Sigma-Aldrich	Cat. #S6508
PMSF	Sigma-Aldrich	Cat. #78830
Protease inhibitor cocktail	Sigma-Aldrich	Cat. #P8340
PhosSTOP phosphatase inhibitor cocktail	Roche	Cat. #04906845001
DTT	Roche	Cat. #10708984001
4x Laemmli Sample Buffer	Bio-Rad	Cat. #1610747
SERVAGel TG PRiME gel	SERVA serving scientists	
PVDF membrane	Millipore	Cat. #IPVH304F0
Nonfat milk	Rapilait	
Bovine serum albumin (BSA)	PAN-Biotech	Cat. #P06-1391100
Tween 20	Sigma-Aldrich	Cat. #8.22184.1000
Anti-Mouse IgG, HRP-linked whole Ab (from sheep)	Amersham	Cat. #NA931V
Anti-Rabbit IgG, HRP-linked whole Ab (from donkey)	Amersham	Cat. #NA934V
Rabbit Anti-Goat Immunoglobulins/HRP	Agilent Dako	Cat. #P044901-2
**Antibodies**		
Rabbit monoclonal anti-iNOS	Cell Signaling Technology	Cat. #13120
Rabbit monoclonal anti-Phospho-p44/42 MAPK (Erk1/2) (Thr202/Tyr204)	Cell Signaling Technology	Cat. #4377
Rabbit monoclonal anti-p44/42 MAPK (Erk1/2)	Cell Signaling Technology	Cat. #4695

Rabbit monoclonal anti-Phospho-CaMKII (Thr286)	Cell Signaling Technology	Cat. #12716
Rabbit polyclonal anti-CaMKII (pan)	Cell Signaling Technology	Cat. #3362
Rabbit monoclonal anti-Phospho-CREB (Ser133)	Cell Signaling Technology	Cat. #9198
Rabbit monoclonal anti-Phospho-NF-κB p65 (Ser536)	Cell Signaling Technology	Cat. #3033
Rabbit polyclonal anti-Phospho-Stat1 (Tyr701)	Cell Signaling Technology	Cat. #9171
Rabbit polyclonal anti-Stat1	Cell Signaling Technology	Cat. #9172
Mouse polyclonal anti-IL-1 beta	RD Systems	Cat. #AF-401-NA
Rabbit polyclonal anti-NFκB p65	Santa Cruz Biotech	Cat. #sc-372
Mouse monoclonal anti-Caspase-1 (p20)	Adipogen	Cat. #AG-20B-0042
Rabbit polyclonal anti-Asc	Adipogen	Cat. #AG-25B-0006
Rabbit polyclonal anti-Pan Actin	Cytoskeleton	Cat. #AAN01
Rabbit polyclonal anti-β-Actin	Cell Signaling Technology	Cat. #4967S
**Solutions**		
Potassium chloride (KCl) solution	Sigma-Aldrich	Cat. #60142
Sodium chloride (NaCl) solution	Sigma-Aldrich	Cat. #71386
Magnesium chloride ($MgCl_2$) solution	Sigma-Aldrich	Cat. #M1028
HEPES solution	Sigma-Aldrich	Cat. #H0887
Calcium chloride ($CaCl_2$) solution	Amresco	Cat. #E506
**Quantitative RT-PCR**		
RNA Lysis Buffer	Zymo Research	Cat. #R1060-1-100
Dithiothreitol (DTT) solution	Sigma-Aldrich	Cat. #43816
Random Primers	Promega AG	Cat. #C1181
dNTP Mix	Promega AG	Cat. #U1515
RNasin Plus RNase Inhibitor	Promega AG	Cat. #N261B
iTaq Universal SYBR Green Supermix	Bio-Rad Laboratories	Cat. #1725121
**Agarose gel electrophoresis**		
Agarose Tablets	Bioline	Cat. #BIO-41027
RedSafe™ Nucleic Acid Staining Solution	iNtRON Biotechnology	Cat. #21141
**Flow cytometry**		
Normal RAT serum (NRS)	Dako	Cat. #X-902
Normal Syrian Hamster Serum	Jackson ImmunoResearch	Cat. #007-000-120
Monoclonal anti-CD11b, PE-Cyanine7	eBioscience	Cat. #25-0112
Monoclonal anti-CD86, APC-R700	BD Biosciences	Cat. #565479
**Assay kits**		
ELISA MAX™ Deluxe Set Mouse IL-1β	Biolegend	Cat. #432604
ELISA MAX™ Deluxe Set Mouse IL-6	Biolegend	Cat. #431304
ELISA MAX™ Deluxe Set Mouse TNF-α	Biolegend	Cat. #430904
Griess Reagent System	Promega AG	Cat. #G2930
Pierce™ BCA Protein Assay Kits	ThermoFisher Scientific	Cat. #23227
RNA MicroPrep kit	Zymo Research	Cat. #R1050
SuperScript III Reverse Transcriptase Kit	ThermoFisher Scientific	Cat. #18080044
CellTiter-Glo Luminescent Cell Viability Assay	Promega AG	Cat. #G7571
**Calcium imaging assay**		
Black, glass-bottom 24-well plate	Greiner Bio-One	Cat. #662892
Fluo-4, AM	Invitrogen	Cat. #F14201
**Other**		
Acetone	Merck	Cat. #100658
DSS	ThermoFisher Scientific	Cat. #21555
Dulbecco's Phosphate Buffered Saline (PBS)	Sigma-Aldrich	Cat. #D8537

Hanks' Balanced Salt Solution (10X)	ThermoFisher Scientific	Cat. #J67771.AP
Opaque-walled white 96-well plate	Greiner Bio-One	Cat. #655073
Paxilline	Tocris Bioscience	Cat. #2006
Iberiotoxin	Tocris Bioscience	Cat. #1086
NS-11021	Alomone labs	Cat. #N-335
